# The Contribution of BaTiO_3_ to the Stability Improvement of Ethylene–Propylene–Diene Rubber: Part II—Doped Filler

**DOI:** 10.3390/polym15163441

**Published:** 2023-08-17

**Authors:** Traian Zaharescu, Alina Dumitru, Tunde Borbath, Ioana Ionescu, Istvan Borbath, Tiberiu Francisc Boros

**Affiliations:** 1 INCDIE ICPE CA, 313 Splaiul Unirii, 030138 Bucharest, Romania; alina.dumitru@icpe-ca.ro; 2 ROSEAL SA, 5A Nicolae Balcescu, 535600 Odorheiu Secuiesc, Romania; borbathistvan@roseal.eu (I.B.); boros.tibor@roseal.eu (T.F.B.); 3 ECOIND, 57-73 Drumul Podu Dambovitei, 060653 Bucharest, Romania; ioana.ionescu@incdecoind.ro

**Keywords:** ethylene–propylene–diene rubber, doped barium titanate, thermal and radiation stabilities, chemiluminescence, thermal analysis, scanning electron microscopy

## Abstract

The thermal and radiation stabilities of the formulations based on ethylene–propylene–diene rubber (EPDM), which contain barium titanate (BaTiO_3_) doped with lanthanum and cerium oxides, were investigated by chemiluminescence and mechanical testing. The contributions of these doped fillers are related to the surface interaction between the structural defects (doping atoms, i.e., lanthanum and cerium) implanted in the filler lattice and the molecular fragments formed during the progress of degradation. These composite materials present extended durabilities with respect to the references; the oxidation periods are a minimum of three times longer than the corresponding times for pristine polymers. This behavior is associated with the scavenging activity of dopants. Mechanical testing has demonstrated the contributions of doped filler to the improvement of tensile strength and elongation at break by the restructuration of the polymer phase. Scanning electron microscopy images revealed the densification of materials in the presence of doped barium titanates. All the investigations constitute valid proof for the qualification of BaTiO_3_ doped with Ce as the more efficient stabilizer compared to the same inorganic filler doped with La.

## 1. Introduction

The main purpose of any manufacturer is the extension of durability for their own products. This goal is achieved by means of several procedures: crosslinking [1,2], compounding [3,4], preparation of nanocomposites [5,6], and stabilization by antioxidants [7,8]. All of these procedures are based on the availability of polymer materials for molecular and morphologic reconstruction, starting from the recombination of fragments. A second phase (inorganic filler) in the polymer substrates interacts with mobile units like radicals, whose identities become attached onto the parent backbones or are recombined with each other to build up new configurations with similar pristine structures. The evolution of stability in the aging products depends, to a large extent, on the contribution of additive or filler to the progression or the mitigation of oxidation. The main factor that influences this behavior is the particle surface of powder, where jointing radicals may be scavenged and withdrawn for different periods from the degrading system. Actually, the surface state, which is characterized by reactive centers like physical or chemical traps, maintains a constant degree of oxidation if the altering units are blocked on it. This situation is learned when the filler is chemically doped [9]. Structural stabilization involves a sudden reduction in the reactivity level of oxidation initiators after their formation. Accordingly, the surfaces of filler particles must be ready for interaction, so that the scavenging centers become active. For the classical action of antioxidants, attraction between the available chemical structures (hindered phenols or amines) and free radicals by their coupling is the required step for the efficient prevention of oxidative degradation [10]. The doped filler, as the highly stable phase in any composite, plays the role of barrier for the development of oxidation, because it slows down the progress of aging by the insulation of oxidizing units from the reactive environment. The composites where the inorganic phase plays the role of stabilizer may be considered as appropriate examples for the extension of operation areas [11].

The mechanistic approach of inorganic phase/polymer nanocomposites offers several ways through which these additives determine the widening of material durability levels under energetic stressing conditions (e.g., thermal, radiation, photo degradation). For example, inorganic complexes inactivate the oxidation of polymer fragments by their coupling with central metallic atoms [12]. Other fillers with spatial geometry, like polyhedral silsesquioxanes, delay degradation due to their cage effect when oxidizing entities penetrate the broken edge separating them from the domains subjected to aging by diffused oxygen [13]. The new lattices structured by doping are suitable versions for attaining the foreseen stability during the application of radiation processing [14]. This innovative idea is applied in this paper: the filler is properly modified for the implementation of radiation technology into the fabrication of high-tech materials.

The presence of doped filler in a polymer substrate brings about a solid-state interaction between the traps that exist inside the first outer layers of the lattice and the free polymer fragments, which have unpaired electrons. Accordingly, they are able to fill the electric gap of superficial defects [9]. This electrical coupling is sufficiently strong, and, consequently, the high energy required for the liberation of scavenged radicals delays oxidation. Thus, the lattice of solid particles becomes the proper compositional modification of long life products, where the initiators of degradation, the native radicals or peroxyl units are intimately caught.

The concentration of traps acting during antioxidative protection is an essential parameter that characterizes the progress of material aging. While the oxidation of basic polymers takes place by the attack of molecular oxygen on weaker bonds in the polymer macromolecules [15], the filler withdraws the radical that is susceptible to oxidation. The relation between the rate of oxidation and the available amount of reacting moieties is based, to a certain extent, on the involvement of deep traps and the sizes of the units to be protected.

In the first part of this series, the stabilization effects of pristine barium titanate were analyzed. The extensive improvement of material strength requiring this type of filler as an appropriate component for several peculiar formulations becomes the milestone for the present investigations. The filler which was utilized, barium titanate, allows for the incorporation of several foreign atoms, forming lattice defects [16]. The fabrication of polymer nanocomposites, including doped barium titanate, was previously reported [17] when the thermal stability was investigated by DSC. If, although the organic substrate, poly(methyletacrylate), may be strengthened by microwave treatment, the contribution of doped titanate is evidently highlighted by the augmentation of T_g_ values as the result of crosslinking. The minimization of polymer aging was also obtained for polyimide nanocomposites containing barium titanate doped with lanthanum [18]. However, a relevant feature related to the energetic involvement of barium titanate nanocomposites involves the storage capability of energy and the association of this filler with the protective ability of polymer substrates [19].

The high-performance polymer nanocomposites based on barium titanate are attained by advanced piezoelectric properties, which involve electrical interactions of the basic material with the surrounding substrate [20]. The homogenous dispersion of filler in the polymer matrix creates a favorable achievement in terms of the localization of energy deposits and an efficient interface between the doping spots and the organic component [21].

The present paper presents the characterization of stability by the effects of doping with lanthanum and cerium atoms in a lattice of barium titanate particles, which act as stabilizer factors in EPDM. These compositions are specially designed for the fabrication of gaskets and O-rings for the safe operation of cooling systems in nuclear power stations. The extension of application ranges places these formulations in the advanced positions of raw materials for the manufacturing of long-term products like window sealing, roof tiles, anticorrosive sheets and layers, and repaired scaffolds. The great advantage of these composites is their improved durability, which is obtained by the contribution of superficial traps from the filler particles. Actually, the main characteristic of stability is particularly based on the chemiluminescence measurements, which offer suggestive images regarding the progress of oxidation and the magnitude of effects produced by the doped barium titanate.

Ethylene–propylene–diene monomer, the polymer substrate used for the preparation of the investigated composites, is an example for many other polymers that may be easy converted into interesting composites. This study represents an additional attempt to demonstrate the stabilization activities of doped inorganic compounds, a new procedure by which several polymers, for example, EPDM, may obtain greater durability. For several years, this improvement method has been reported [22,23] as a versatile method for the extension of the lifetimes of polymer materials, where the classical antioxidants, hindered phenols and amines, are efficiently replaced by inorganic compounds with modified lattices. They are appropriate versions to be used for the manufacturing of high-performance materials subjected to accelerated degradation by thermal and radiation aging.

## 2. Materials and Methods

The present formulations of composites were previously characterized when the filler was barium titanate as raw material [23].

### 2.1. Materials

Ethylene–propylene–diene monomer, manufactured and supplied by DSM Elastomers (Heerlen, The Netherlands) as KELTAN 8340, was the polymer substrate used for the preparation of the investigated composites. The pristine polymer material contained ethylene and propylene components in the proportion of 3:1. A certain amount (5 wt%) of 5-ethylidene 2-norbornene (EBN) was also present in the polymer structure. Raw barium titanate (Shandong Deshang Chemical Co., Ltd., Jining, China) was modified by doping with cerium and lanthanum oxides. The general formula for the resulting powders after their thermal treatment of inclusion was Ba_1−x_M_x_TiO_3_, where M was La^3+^ or Ce^4+^. The doping molar fraction, x, was 10^−3^. The raw materials, BaTiO_3_, La_2_O_3_, and CeO_2_, were pro-analysis grade. The last two materials were purchased from Sigma Aldrich (St. Louise, MO, USA). The initial materials were ground for 10 h under wetting with ethylic alcohol in an agate crucible by means of agate balls. After attaining an average size of 100 μm, the powders were dried in an electrical oven at 80 °C until the alcohol smell disappeared. The incorporation of each powder into the polymer was carried out by mixing the components in a homemade thermoforming roller unit with a capacity of 1 kg for the homogenization of the powder distributions. The concentrations of the doped fillers were 1 phr and 2.5 phr.

### 2.2. Methods

After the preparation of pristine formulations, some plates with thicknesses of 2 mm were pressed in a specific machine produced by Nicovala (Sighisoara, Romania). All details were previously reported [23]. The γ-processing was accomplished in an irradiator provided with ^60^Co source (Ob Servo Sanguis, Budapest, Hungary) in air at room temperature. The optimal dose rate applied during irradiation was 0.5 KGy h^−1^. The processing doses were 0, 50, and 100 kGy.

The modifications induced by γ-irradiation and thermal degradation were investigated by chemiluminescence (CL), mechanical testing, and scanning electronic microscopy (SEM). The CL spectra were recorded as the two types of determinations: nonisothermal measurements at the heating rate of 15 °C min^−1^ and isothermal regimes at 180 °C, a convenient value for the appropriate rates of oxidation. The normalization of emission intensities to the unit mass allows for a comparison of results that illustrates the correct differences between various samples. These procedures are described in detail in an earlier paper [24]. The details on mechanical testing were presented in the first part of this series [23].

Mechanical testing was conducted using the ZMR 250 equipment (VEB Thuringer, Raunestein, Germany), applying the testing standard ISO 37/2012 [25]. The evaluation of Shore A hardness was based on ISO 7619/1 (2011) [26] with testing unit equipment purchased from Stendal, Stendal, Germany.

The microscopic investigations were carried out using the scanning electron microscope Quanta 250 FEG (field emission gun) from FEI (Thermo Fisher Scientific, Mundelein, IL, USA). The microstructure investigation was accomplished with the most versatile high-resolution SEM provided with an FEG field emission electron source. The assays were carried out in LoVac (low vacuum) working mode under a working tension of 10,000 V. The image magnitude is around 2200×.

## 3. Results

**Figure 1:**
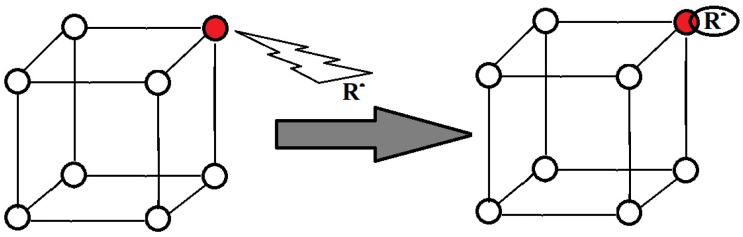
The schematic principle by which the free radical formed by the scission of macromolecules during degradation is tightly attached to the lattice gap. Red circle indicates the doping atom, which scavenges the degradation intermediate R.

This behavior analysis is a demonstration of the capability of doped inorganic materials for the improvement of polymer durability, while this class of materials is subjected to an advanced oxidation process. This proposed manner of stabilization was previously checked [22,23] as a versatile replacement of organic antioxidants that are less unstable and unhealthy. The inevitable exposure where exceeding energetic transfer occurs, for example, the photodegradation induced by sunlight or the radiochemical aging, produces major structural modifications. The forced or accidental high-energy irradiation applied for the processing or qualification of polymer products in various installations (electron beam accelerators, γ-irradiators, or nuclear reactors) are relevant cases of initiated degradation, where the material may exhibit new and unforeseen comportment during the operation periods if a suitable antiaging agent is absent. Figure 1 presents the principle on which this paper was built.

### 3.1. Chemiluminescence

The advancement of oxidative degradation occurs when the oxygen penetrates the polymer matrix. The accumulation of hydroperoxides that are formed according to the classical mechanism reported by Bolland and Gee [24,27] is the main way in which the competition between the material crosslinking and degradation characterizes the progress of aging. As was experimentally stated in an earlier paper [28], there is a dose threshold before the initial crosslinking of EPDM takes place where the rate of recombination exceeds the degradation rate. Then, the higher concentration of free radicals may be a main reason for oxidation promoting the worsening of the product.

**Figure 2:**
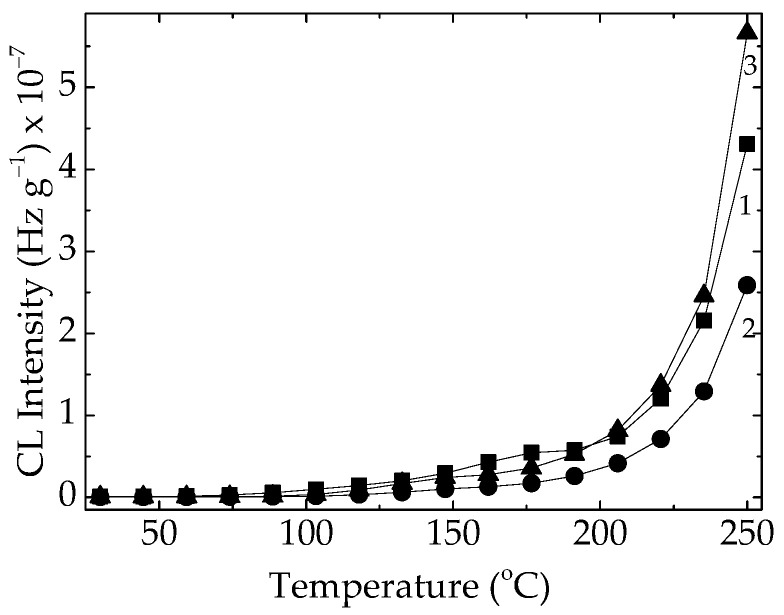
Nonisothermal CL spectra recorded on pristine material subjected to tree exposure γ-doses: (1) 0 kGy; (2) 50 kGy; and (3) 100 kGy. Heating rate: 15 °C min^−1^.

The pristine EPDM containing various amounts of unsaturation (diene units) is simultaneously subjected to molecular scission and crosslinking. Figure 2 illustrates the improvement of elastomer stability due to the increase in onset oxidation temperatures from 175 °C to 208 °C, indicating a real strengthening caused by γ-irradiation.

**Figure 3:**
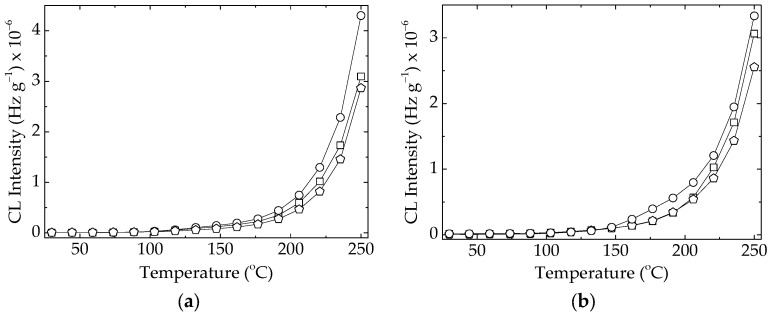
Nonisothermal CL spectra recorded on the unirradiated EPDM composite samples in the presence of various fillers. Heating rate: 15 °C min^−1^. Barium titanate concentration: (**a**) 1 phr; (**b**) 2.5 phr; (circle) neat filler; (square) filler modified with lanthanum; (pentagon) filler modified with cerium.

**Figure 4:**
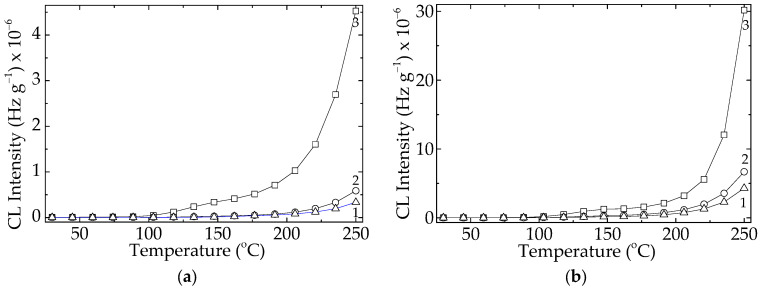
Nonisothermal CL spectra obtained for the radiation processed EPDM/modified BaTiO_3_ samples with 2.5 phr of filler. Heating rate: 15 °C min^−1^. Lattice modified with (**a**) cerium and (**b**) lanthanum. Exposure dose: (1) 0 kGy; (2) 50 kGy; (3) 100 kGy.

The presence of dopant, either lanthanum or cerium, in the composition of the filler improved the thermal resistance of the polymer at the both levels of loadings (Figure 3). Experimentally, it may be noted that the measured values of CL intensities for these specimens are lower than the figures recorded in the cases of pristine polymer (Figure 3). On the other hand, the effect brought about by lanthanum is inferior to the contribution provided by cerium dopant. This behavior was emphasized in another previous paper [9], where the electron density of the dopant was shown to play a decisive role in the stabilization activity of lead titanate (Figure 4).

**Figure 5:**
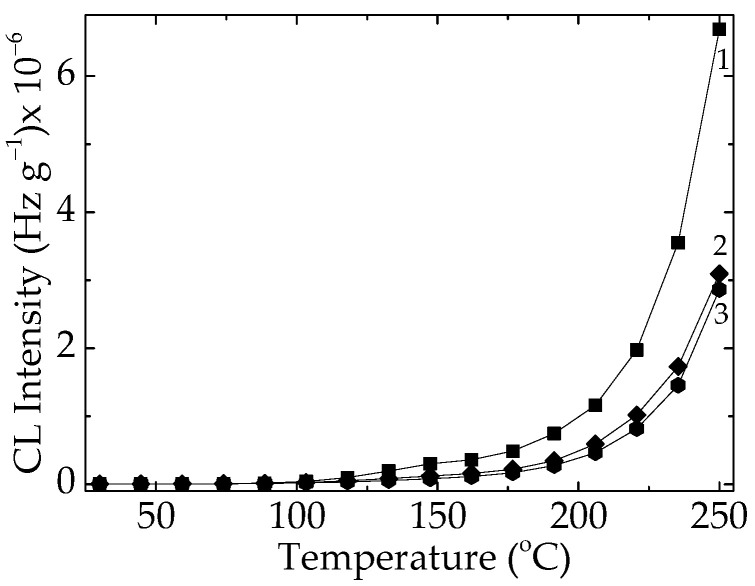
Nonisothermal CL spectra drawn for the EPDM composites irradiated at 50 kGy. Filler content: 1 phr. Heating rate: 15 °C min^−1^. Barium titanate filler: (1) pristine material; (2) compound modified with La; (3) compound modified with Ce.

The diminution of the destructive effects caused by ionizing radiation regarding the protection of polymer material was also revealed [29,30] by the mitigation of energy transmission through the sheet of polymer nanocomposites. Other stabilization proofs sustaining the beneficial addition of titanate in the formulations of compositions are offered by the behavior and properties of several materials with various destinations, i.e., dielectric features of insulators [31], fabrication of sensors [32], and electrical engineering at high service temperatures [33]. These applications are possibly due to the intimate interaction between filler and polymer that keeps the two phases closely together. If the curves drawn for the lower concentration (1 phr) of filler are somewhat well separated (Figure 3), the development of oxidation in the EPDM probes overlaps until the temperature reaches 205 °C. This means that the higher loading of doped contents sweeps the difference that exists for the smaller amounts of lattice defects. When the studied compounds are accidentally aged during their stressing by an overcharge effort, the different levels of stabilization activities shown after γ-irradiation due to the accelerated degradation are obtained. For the dose of 100 kGy a high local concentration of radicals may be supposed, and more efficient protection by BaTiO_3_/Ce than BaTiO_3_/La (Figure 5) is obtained.

Even at a moderate irradiation dose (50 kGy) (Figure 5), both doped versions of Ba TiO_3_ are suitable inhibitors against oxidation. The onset oxidation induction time is 178 °C for neat titanate, while for doping by lanthanum and cerium, this occurs at 197 °C and 208 °C, respectively. The contributions of doping elements are related to their different electronic densities, as efficient gaps exist where unpaired electrons of free radicals find proper places to be coupled [34].

**Figure 6:**
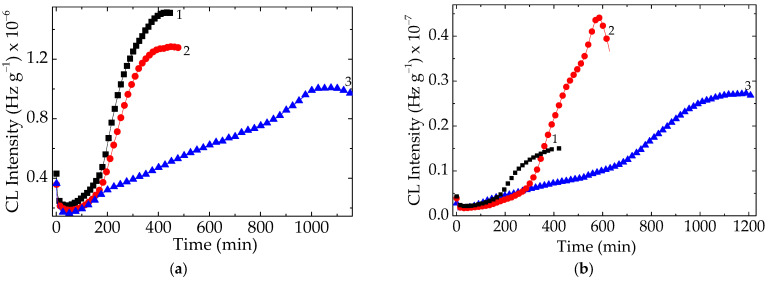
Isothermal CL spectra recorded on the EPDM under various doping states of titanate. Filler concentration: (**a**) 1 phr; (**b**) 2.5 phr. Testing temperature: 180 °C. Sample composition: (1) EPDM+ neat titanate; (2) EPDM + titanate modified with La; (3) EPDM + titanate modified with Ce.

The isothermal chemiluminescence measurements which present the evolution of oxidation over time also reveal the high efficiency of stabilization brought about by cerium doping in barium titanate (Figure 6), as was previously proven [22]. If the growth of stability is smooth, when lanthanum atoms exist in the lattice, the presence of cerium extends the oxidation period by two times and a half.

The increase in the filler loadings, particularly the doped titanate, associated with the radiation processing by high energy exposure to γ-rays promotes advanced improvement in the material stability, which has been proven by the evolution of oxidation. The values of oxidation induction time (OIT)—the moment when the degradation theoretically starts—increased from 135 min, 182 min, and 190 min for unmodified, La-modified, and Ce-modified titanates to 186 min, 288 min and 620 min, respectively (Figure 6). This represents a real gain in terms of the durability of products manufactured using this technological procedure. The main modification was obtained with the samples containing 1 phr of barium titanate doped with cerium (Figure 6a,b).

The comparison between the CL spectra recorded for EPDM composites containing doped barium titanate reveals several characteristics that recommend them as an efficient type of oxidation protector in a large category of polymers [35,36]. However, there are very few published papers which are dealing with the consequences of doping fillers on the modification of polymer stability [37]. Thus, the evaluation of the composition factors and their effects the durability performances of polymer composites is still awaiting a new assay.

**Figure 7:**
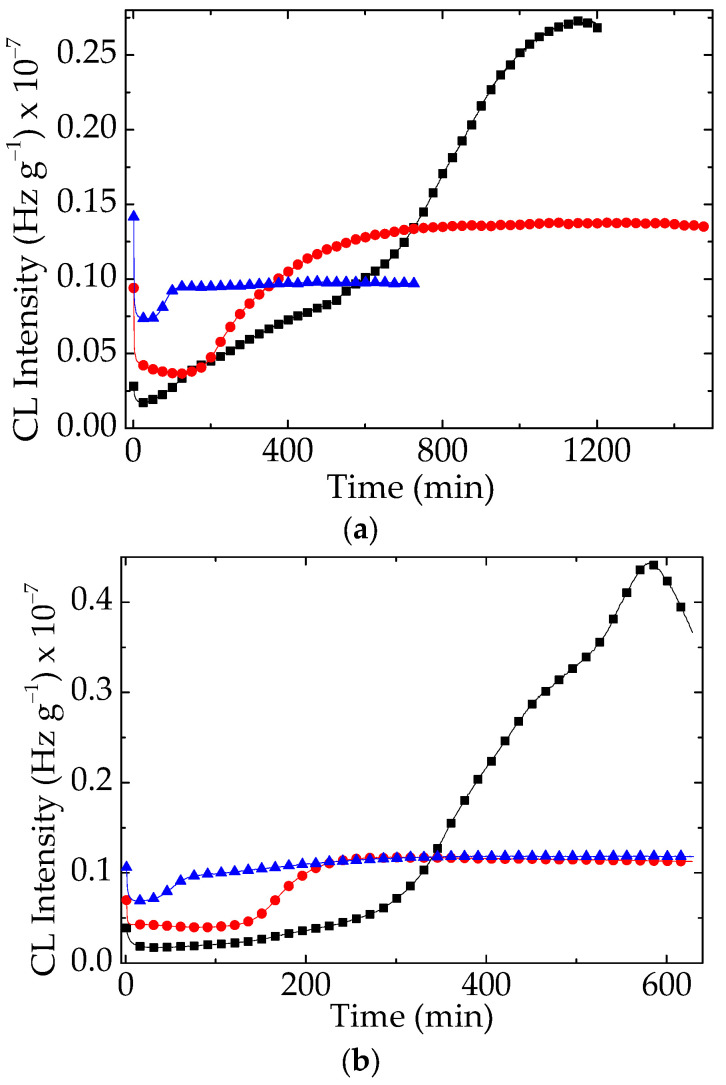
Isothermal CL spectra obtained on the EPDM/modified BaTiO_3_ composites. Testing temperature: 180 °C. (**a**) BaTiO_3_/La; (**b**) BaTiO_3_/Ce. Irradiation dose: (black square) 0 kGy; (red circle) 50 kGy; (blue triangle) 100 kGy.

As is demonstrated in Figure 7, the γ-exposure improved the durability of EPDM substrate because the radicals jointing the particle surface were released and involved in recombination with other available radicals.

The most interesting aspect of the CL curves recorded on the γ-irradiated materials is the presence of a pseudo-plateau in the propagation stages. This suggests that this radiochemical treatment creates additional defects in the titanate crystal lattice, which increases the protection activities of inorganic phase as it occurs; a stable and efficient antioxidant ensures the delay of degradation [38].

The addition of titanate filler into the polymer-based products is a main factor that allows the evolution of the aging of the polymer matrix to lead to long-term durability, as was earlier demonstrated in a previous paper in this series [22].

As was reported [9] previously, the presence of inorganic structures in the composition of polymers indicates a pertinent improvement of stability, even in the materials processed at advanced levels of irradiation.

The evolution of CL curves shown in Figure 7 highlights the contribution of doped filler, which minimizes the oxidation rates during the propagation step. The proof of this effect on the delay of oxidation is the presence of a pseudo-plateau that keeps the generated hydroperoxides constant.

### 3.2. Mechanical Testing

The mechanical behavior of these studied composites reflects the contribution of an interphase interaction that keeps the filler tightly together in the polymer environment (Table 1). The incorporation of various doping forms of barium titanate indicates not only the modification of thermal strength previously evaluated by chemiluminescence (Section 3.1), but also the mechanical behavior. The discontinuity of the investigated materials consisting of two unlike phases determines some significant differences between the mechanical features (elongation at break and tensile strength) due to the disruption of intermolecular interactions. However, the γ-irradiation and the contributions of doped fillers to the modeling of thermal stabilization have behavioral consequences on the studied polymer as it relates to the mechanical charge. While the unirradiated composites which were present improved the values for elongation at break, as well as for the tensile strength, the exposure at 50 kGy induced better concern by the filler doped with cerium than that with the involvement of lanthanum. For both irradiation doses applied for the sample processing, the increase in the filler concentration significantly ameliorated the mechanical features of the composites. Even at 100 kGy, the quality of the processed formulations was significantly enhanced, because the doped structures assisted in the augmentation of the materials’ stabilities.

The values of hardness are not suggestively modified. They do not become the characteristic property due to small differences in the local intermolecular strength. Although the growth of stability would be promoted by increased crosslinking, this augmentation is enough for the protection against oxidation, but it is not sufficient for the modification of material hardness.

### 3.3. Scanning Electron Microscopy

The structural images of the present polymer composites are shown in Table 2. They illustrate the cumulative effects of compounding and γ-irradiation on the material consistencies. While high-energy exposure produces molecular scission, the filler acts as a protection agent. The microstructures of the samples containing modified barium titanate revealed that they developed crosslinking, which is possible due to the scavenging of free radicals prior to their oxidation reactions. The densification of materials is the natural consequence of the involvement of defects existing on the particles’ surfaces after the insertion of the doping elements, i.e., cerium and lanthanum. The inclusion of a modified filler brings about the possibility of separating the reactive radicals from the surrounding polymer. The direct outcome of radiation processing of EPDM/BaTiO_3_ composites is the correlation between the protection activity promoted by the filler and the possible recombination of intermediates starting from the compositional presence of diene, ENB (5-ethylidene-2-norbornene) [39]. The inorganic component would play the role of barrier in the diffusion of oxygen. Consequently, a slight diminution of the local content of oxygen allows for the propagation of crosslinking, where the participants are supplied by the particle surface through detachment. The comparison between the effects of the three kinds of barium titanate used in this study points out the larger contribution of doped filler to the compaction of the organic phase with respect to the pristine titanate.

The micrographs of EPDM/BaTiO_3_ composites suggest that the inorganic phase contributes to the achievement of a longer life, as the aging of the material would force the structures to be damaged, with direct consequences on durability. The presence of these compounds is emphasized by the gradual strengthening under γ-irradiation (Table 2).

## 4. Discussion

The analysis of peculiar contributions of doped barium titanate to the stability of EPDM as a representative for polymer materials highlights the significant ability of the filler to insulate of free radicals by superficial scavenging them from the oxidizing spots during the propagation stage of degradation. This activity defines the coupling of lattice defects consisting of the presence of dopants with different electron densities. The conversion of free radicals into stable structures is the effect of the interaction between these units and the traps from the surface of filler particles, where the electron gaps attract the unpaired electrons of radicals and, consequently, the molecular fragment carriers. Consequently, the progress of oxidative degradation is slowed down according to the electronic interactions between the close entities that attract each other [40].

The inclusion of doped barium titanate in the compositions of structured materials leads to extended life, which has been demonstrated by the kinetic parameters of thermal degradation as well as by the suggestive images recorded using the SEM instrument. Our attention was mainly paid to the long oxidation times (Figure 7) achieved due to the better values of tensile strength and elongation at break. They indicate the participation of the filler in the breaking propagation chain of oxidation. In contrast with the pristine EPDM, the composites are characterized by the augmentation of their stabilities, when the concentration of doped titanate becomes higher from 1 phr to 2.5 phr (Figure 3). The viability characterization of our compositions is based on interconnection between the two phases, which have a functional assembly, like homogeneous blends [41]. The vicinities of radicals and inorganic lattices would stimulate the association between the polymer fragments and structural “defects” like doping atoms, allowing for tight scavenging by the unpaired electrons of degradation intermediates [42]. In spite of the lack of information on the stability of EPDM/titanate, the doped filler is a promising component that makes the fabrication of long life products with prominent performances possible [43,44,45].

Usually, these kinds of composite are studied as high-tech materials with piezoelectric applications. Though significant attention is paid to their physical properties, for example [46], the improved behavior related to the delay of oxidation was not still remarked. The present results, focused on the filler contributions to the inhibition of aging (Figure 6), indicate the appropriate route through which several technologies are capable of providing highly resistant items, including spare parts for nuclear engineering and aircrafts. The polymer dielectrics which operate by electronic interaction are illustrative examples of the contribution of the inorganic salt phase to the integrity and stabilization suitability of products [47].

**Figure 8:**
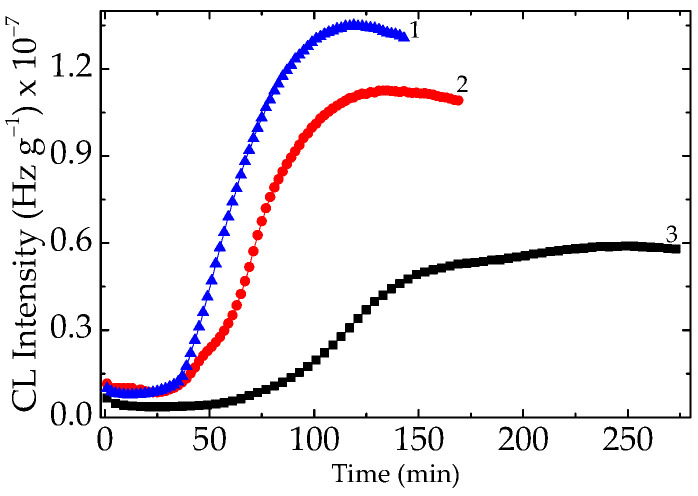
The isothermal CL recorded on unirradiated EPDM/BaTiO_3_/Ce, carried out at 200 °C. Filler loading: (1) 0 phr; (2) 1 phr; (3) 2.5 phr.

As was reported in an earlier part of this series [22], the evaluation of the thermal and radiation strengths of EPDM/BaTiO_3_ is sustained by trapping radicals in the superficial gaps. This must not be considered as an antioxidant feature, considering that the mechanisms are completely different [9,48]. The evaluation of the thermal and radiation strengths of EPDM/BaTiO_3_ is sustained by trapping radicals in the superficial gaps, because this characteristic involves the substitution of protons. However, the physical attraction is strong enough that is illustrated by the very long duration of chemiluminescence determination at high temperatures. The nonisothermal measurements of the evolution of oxidation indicate these high temperatures as the starting points for the initiation of the process (Figure 4), even at a moderate exposure dose (50 kGy). This characteristic feature is related to the antirad action, which emphasizes proper radiation resistance over long operation periods [49]. The slow evolution of oxidation, either in unirradiated or radiation-processed samples containing doped filler, is a useful indication of the intrinsic participation of the titanate filler. The additional CL measurements (Figure 8) carried out at 200 °C reveal the efficiency of this compound with respect to the accidental increase in operation temperature.

As a corollary of the presented results, the stabilization activities of doped BaTiO_3_ are principal characteristics that indicate the potential versions available for the improvement of polymer durabilities under energetic stress conditions.

## 5. Conclusions

This paper presents conclusive results on the peculiar contributions of doping to the stabilization activity of barium titanate as an investigative proof of the stabilization action of electronic gaps in the lattices of inorganic crystals. The improvement noticed in the kinetic parameters during the thermal and radiation aging (onset oxidation temperature and oxidation induction time), as well as the longer duration of thermal degradation of ethylene–propylene–diene monomer (composites are the relevant routes for real options in the manufacturing technologies of polymer materials). The extension of oxidation duration by several times, the slow oxidation rates during the propagation of oxidation, the compaction of sample microstructures, and the improvement of mechanical properties (elongation at break and tensile strength) are the consequences of the stabilization activities of doped barium titanate on the oxidation strength of a hydrocarbon polymer, EPDM. The addition of these fillers (BaTiO_3_ doped with cerium and lanthanum) causes oxidation to be delayed by the inactivation of reactivity during oxidative aging due the trapping of radical fragments on the lattice defects formed in the first outer layers of the inorganic particle surface. These results prove that inorganic structures may help the aging polymers to delay oxidation if the environmental or operation conditions were to reach proper parameters of degradation.

This paper fills an existing gap in the study of valid compounds that can promote an efficient increase in polymer stability when subjected to accelerated degradation. It recommends the substitution of classical organic antioxidants whose structures are more easily modified under accelerated damage. The doped BaTiO_3_ is an appropriate solution for the manufacturing of long-life items, because the propagation chain of oxidation is broken by the joining of free radicals on the particle surface where the doped atoms are placed in the discontinuities of the titanate lattice.

The presence of barium titanate as a suitable filler whose main purpose is improving the lifetimes of materials suggests an adequate solution to be applied to several polymers for their extended warranties under severe activity conditions.

These results open a new route for the production of high-durability polymer materials, which may be used in a large category of applications where highly resistant stability support is mandatory.

## Figures and Tables

**Table 1:** Summarized values of mechanical testing achieved on the EPDM composites with doped BaTiO_3_.

EPDM Composition	Elongation at Break (%)	Tensile Strength (daN mm^−2^)	Hardness (Shore A)
Dose: 0 kGy			
Neat EPDM	940 ± 98.4	4.36 ± 1.25	60.8 ± 0.86
1 phr of pristine BaTiO_3_	1190 ± 99.9	8.05 ± 1.19	64.0 ± 1.20
2.5 phr of pristine BaTiO_3_	1403 ± 130.7	11.44 ± 1.28	61.7 ± 1.20
1 phr of La-doped BaTiO_3_	1307 ± 103.7	10.34 ± 1.26	64.8 ± 1.19
2.5 phr of La-doped BaTiO_3_	1053 ± 93.8	6.11 ± 1.02	64.7 ± 1.21
1 phr of Ce-doped BaTiO_3_	1232 ± 100.7	8.64 ± 1.04	64.5 ± 1.20
2.5 phr of Ce-doped BaTiO_3_	1236 ± 104.5	12.30 ± 1.29	64.5 ± 1.04
Dose: 50 kGy			
Neat EPDM	540 ± 47.3	4.81 ± 1.01	62.2 ± 1.00
1 phr of pristine BaTiO_3_	513 ± 44.6	7.07 ± 1.26	64.0 ± 1.04
2.5 phr of pristine BaTiO_3_	600 ± 44.5	9.92 ± 1.29	63.2 ± 0.99
1 phr of La-doped BaTiO_3_	892 ± 74.7	5.35 ± 1.18	62.2 ± 0.97
2.5 phr of La-doped BaTiO_3_	616 ± 53.4	3.19 ± 1.06	64.0 ± 1.01
1 phr of Ce-doped BaTiO_3_	830 ± 71.8	4.32 ± 1.05	63.2 ± 1.03
2.5 phr of Ce-doped BaTiO_3_	923 ± 80.1	6.36 ± 1.12	63.7 ± 1.05
Dose: 100 kGy			
Neat EPDM	500 ± 42.5	4.05 ± 1.19	64.5 ± 1.09
1 phr of pristine BaTiO_3_	487 ± 40.4	7.03 ± 1.25	63.5 ± 1.04
2.5 phr of pristine BaTiO_3_	500 ± 40.6	6.99 ± 1.25	65.1 ± 1.11
1 phr of La-doped BaTiO_3_	616 ± 55.8	3.19 ± 1.12	64.0 ± 1.08
2.5 phr of La-doped BaTiO_3_	660 ± 59.8	4.45 ± 1.15	64.8 ± 1.07
1 phr of Ce-doped BaTiO_3_	691 ± 59.7	3.84 ± 1.14	64.5 ± 1.10
2.5 phr of Ce-doped BaTiO_3_	743 ± 65,9	5.13 ± 1.21	64.8 ± 1.11

**Table 2:** The micrographs recorded on EPDM/modified BaTiO_3_ composites.

Material	D 0	D 100
EPDM	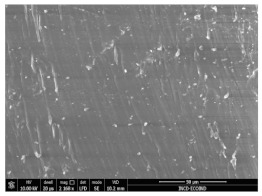	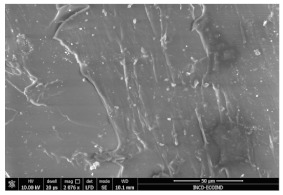
EPDM 2.5	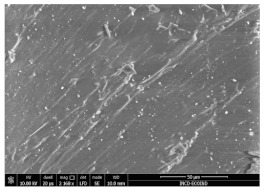	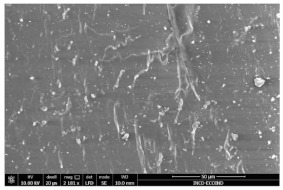
EPDM 2.5 La	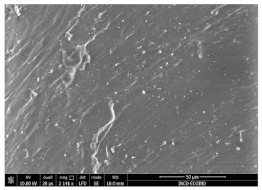	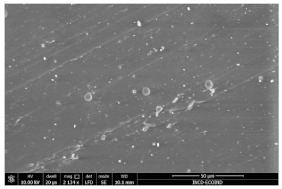
EPDM 2.5 Ce	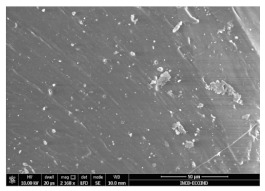	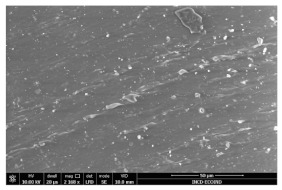

## Data Availability

The data presented in this study are available upon request from the corresponding authors.
